# Clinical Conundrum: Unveiling a Unique Presentation of Hypopharyngeal Carcinoma

**DOI:** 10.7759/cureus.57727

**Published:** 2024-04-06

**Authors:** Anoushka Sahai, Ritika Dixit, Rekha Choudhary, Hetal Marfatia, Prateek Mohapatra

**Affiliations:** 1 Otolaryngology - Head and Neck Surgery, Seth GS Medical College and KEM Hospital, Mumbai, IND

**Keywords:** tobacco chewing, tracheostomy, sideropenic dysphagia, hypopharyngeal carcinoma, subcutaneous emphysema

## Abstract

Dysphagia is a common symptom encountered in clinical practice, typically associated with a wide range of etiologies, including structural abnormalities, inflammatory conditions, neoplasms, and neurological disorders. However, the combination of subcutaneous emphysema, vocal cord palsy, enlarged arytenoids, and pooling of saliva in a dysphagic patient represents a rare and intriguing presentation.

A 33-year-old female presented at a tertiary care hospital in Western India with hoarseness of voice, difficulty in swallowing, productive cough, and neck pain for two months with an abrupt increase in the severity of all symptoms in two days. A history of chewable tobacco use for six years was disclosed. Clinical evaluation revealed a thin build with platynychia and conjunctival pallor, dental staining, drooling of saliva, the presence of extensive subcutaneous emphysema on palpation of the neck, and absent laryngeal crepitus. Endoscopic evaluation was suggestive of right vocal cord palsy and enlarged, congested arytenoid cartilages, post-cricoid growth with pooling of saliva in bilateral pyriform fossae. A CT scan of the neck showed a 2x3 cm neoplastic growth in the hypopharynx, with subcutaneous emphysema and free air foci in the head and neck region, prompting an immediate tracheostomy and biopsy of the hypopharyngeal growth with Ryle’s tube insertion. Squamous cell carcinoma was confirmed on the biopsy report.

Due to its rarity, the possible underlying cause of idiopathic subcutaneous emphysema should be sought whenever encountered in clinical practice since these patients are potentially misdiagnosed. A high index of suspicion among clinicians, along with a consideration of the constellation of other symptoms and clinical features of a possible underlying hypopharyngeal cancer whenever encountering such patients is of key importance for prompting further investigations and treatment.

## Introduction

Dysphagia is a common symptom encountered in clinical practice, typically associated with a wide range of etiologies, including structural abnormalities, inflammatory conditions, neoplasms, and neurological disorders [1,2]. However, the combination of subcutaneous emphysema, vocal cord palsy, enlarged arytenoids and pooling of saliva in a dysphagic patient represents a rare and intriguing presentation.

## Case presentation

A woman in her early 30s had presented at a tertiary care hospital in Western India with hoarseness of voice, difficulty in swallowing, productive cough and neck pain for two months. An abrupt increase in the severity of all symptoms had afflicted her for two days, with a complete inability to swallow both liquids and solids. A history of chewable tobacco use for six years was disclosed. There was no associated fever, weight loss or respiratory distress. General examination revealed a thin built with platonychia and conjunctival pallor. ENT examination showed dental staining, drooling of saliva and presence of extensive subcutaneous emphysema on palpation of the neck, extending from the right cheek to the clavicle and crossing the midline of the neck to the contralateral clavicle. No other cervical lymphadenopathy was present clinically. Laryngeal crepitus was notably absent. Endoscopic evaluation was suggestive of right vocal cord palsy and enlarged, congested arytenoid cartilages, post-cricoid growth with pooling of saliva in bilateral pyriform fossae.

**Figure 1:**
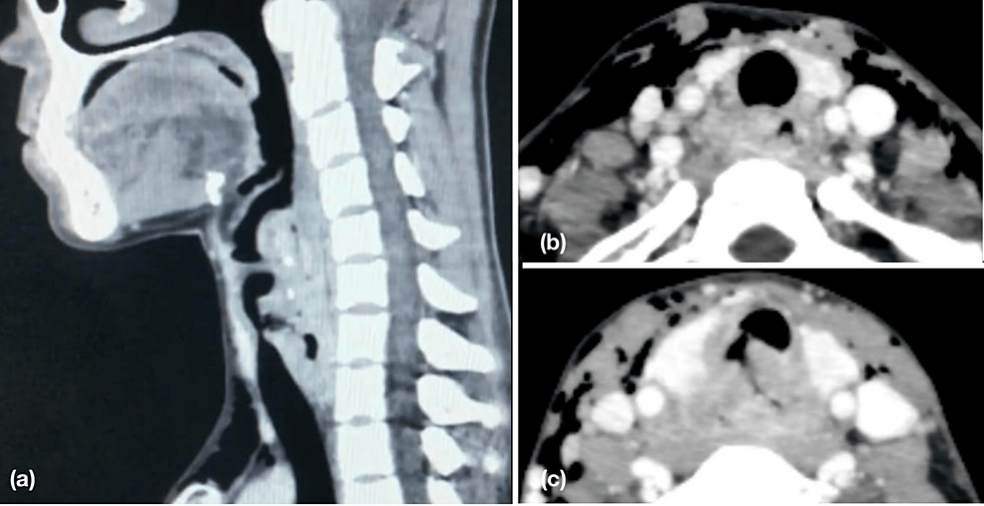
CT neck with contrast revealed an ill-defined heterogeneously enhancing soft tissue epicentered at the hypopharynx measuring 2 * 3 cm with soft tissue thickening extending from C5 to T1 vertebral level in sagittal view (a). The presence of subcutaneous emphysema and prevertebral space involvement in axial view (b). A suspicious communication of the lesion with the posterior wall of trachea and bulging soft tissue at the posterior wall of trachea in axial view (c).

Given the unique combination of clinical findings, diagnostic assessment aimed to identify the underlying etiology with the use of radio-imaging. Contrast-enhanced computed tomography of the neck and chest was performed to identify the cause connecting this myriad of clinical findings, which disclosed a 2x3 cm ill-defined heterogeneously enhancing soft tissue epicentered at the hypopharynx, involving aryepiglottic folds, right pyriform sinus and left true vocal cord with suspicious fistulous communication with the trachea, suggestive of a neoplastic etiology. Subcutaneous emphysema and free air foci were noted in the head/neck region and anterior mediastinum (Figures 1a-1c).

**Figure 2:**
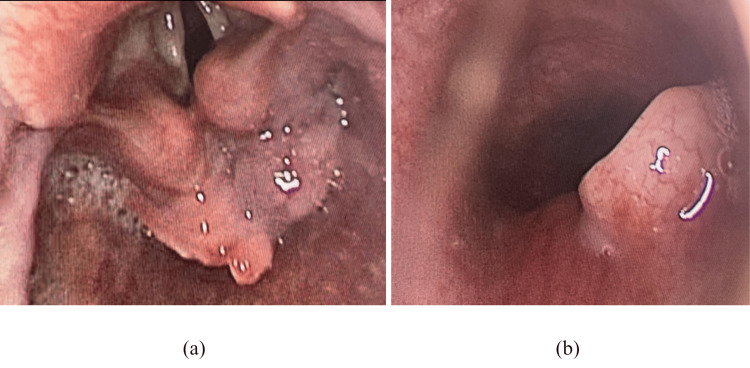
Direct laryngoscopy intra-operatively revealed (a) the presence of ulceroproliferative growth involving the post-cricoid region. (b) An isolated defect in the subglottic region was present due to infiltration by the tumor.

She was taken to the operation theatre by the ENT surgeons for a diagnostic biopsy from the post-cricoid region as well as endoscopic evaluation of the trachea and planning the level of tracheostomy. Intra-operatively, a post-cricoid proliferative growth was present which also involved both the pyriform sinuses. On evaluation with an endoscope, the growth was found to be evading the trachea posteriorly in the subglottic region (Figures 2a, 2b). However, the rest of the trachea was found to be normal. Tracheostomy was undertaken at the level of the third tracheal ring and a portex tracheostomy tube of 6.5 number was placed. This also helped in securing the airway from aspiration. A biopsy was taken of the growth and Ryle’s tube insertion was done to ensure adequate nutrition and the patient’s condition was vigilantly monitored.

Post procedure, the patient and family were educated regarding the tracheostomy care and kept under observation. The biopsy report was confirmatory for moderately differentiated squamous cell carcinoma. The patient was started on concurrent chemo-radiation at the oncology center for the same.

## Discussion

Hypopharyngeal cancer, a formidable health challenge in India, encompasses malignancies occurring within the pyriform sinus, post-cricoid area, and posterior pharyngeal wall. This pervasive threat has manifested itself with a disquieting annual incidence of 75,000 cases, contributing significantly to the demographic landscape of cancer patients in the country, ranging from 5% to 10% of the total [3,4]. Among the diverse histopathological profiles observed, squamous cell carcinoma prevails as the most prevalent, with its genesis intrinsically linked to the consumption of tobacco and/or alcohol [5].

Tobacco chewing, deeply ingrained in the cultural fabric of India, is notably facilitated by its accessible packaging and sophisticated marketing campaigns, exacerbating the susceptibility of individuals to hypopharyngeal cancer. The study conducted by Sapkota et al. [3] delineates the relationship of chewable tobacco with hypopharyngeal carcinoma. This was also corroborated in our case.

Globally, hypopharyngeal carcinoma is more prevalent in males in the middle-aged group [6]. However, in our case report, we highlight a rare case of hypopharyngeal carcinoma in a young female. It can be postulated considering her presentation that sideropenic dysphagia and tobacco consumption had synchronously led to the pathogenesis of the malignancy. Sideropenic dysphagia has been widely linked to iron deficiency which is a common occurrence in Indian women [7,8]. The oxidative stress and DNA damage induced due to malfunctioning of various antioxidant enzymes can lead to metaplastic changes in the epithelia of the hypopharynx and cervical esophagus [5].

The typical clinical exhibition of hypopharyngeal cancer encompasses an array of distressing symptoms, ranging from the visibility of neck masses to lingering sore throats, difficulties in swallowing (dysphagia), the onset of hoarseness in voice, or even upper airway obstructions, contingent upon the specific subsite that is affected [9].

Hypopharyngeal carcinomas are known to be asymptomatic till late stages owing to their intrinsic location. This has led to a substantial proportion of patients (ranging from 70% to 90%) being diagnosed at a stage where the disease has already reached an advanced state (Stage III or IV) [9]. Our patient also sought medical help only after the sudden onset of subcutaneous emphysema in the neck. The literature chronicles only two similar cases where the first presenting symptom is subcutaneous emphysema however both cases pertain to laryngeal carcinoma [10,11]. To our knowledge, this is the first reported case of a patient presenting with subcutaneous emphysema in advanced hypopharyngeal carcinoma in the English literature. This extraordinary rarity underscores the propensity for misdiagnosis and subsequent diagnostic delays that add up to the mortality rate of the entity. The careful clinical examination that helped us in clenching the diagnosis was the presence of laryngeal widening, lack of laryngeal crepitus, and pooling of saliva in bilateral pyriform fossae on endoscopy. The presence of post-cricoid growth is not well appreciated in the rigid office-based sitting 70-degree endoscopy as it is a hidden area and is well appreciated during the direct laryngoscopy procedure [12]. The presence of pooling of saliva in both the pyriform fossae thus is a telltale indicator for evaluation of patients with direct laryngoscopy/ fiberoptic laryngoscopy and esophagoscopy.

The infiltration of the post-cricoid malignancy in our patient to the trachea, and prevertebral space had led to the categorization into stage T4b N0, i.e., Stage IV B (AJCC 8th edition). As an ominous consequence of this tardiness, the prognosis for individuals afflicted by late-stage hypopharyngeal cancer remains dire, with the statistics revealing an estimated five-year survival rate of merely 22% for Stage IV. The involvement of prevertebral space had made our patient non-operable and was considered for concurrent chemoradiation [13,14].

In summation, hypopharyngeal cancer has emerged as an unequivocal public health conundrum in India, its relentless march augmented by an alarming incidence rate and the pernicious partnership it forms with tobacco and iron deficiency. The untimely diagnosis of this insidious malignancy serves as an alarming harbinger of the dire need for heightened vigilance, public awareness campaigns, and innovative diagnostic methodologies.

## Conclusions

The exceptional manifestation of subcutaneous emphysema within the domain of hypopharyngeal carcinomas underscores the intricate and multifaceted nature of this disease and simultaneously highlights the profound implications of diagnostic delays. A high index of suspicion among clinicians, along with the consideration of the constellation of other symptoms and clinical features of a possible underlying hypopharyngeal cancer whenever encountering such patients is of key importance for prompting further investigations Thus, holistic interventions aimed at early detection, and management can reduce mortality burden imposed by hypopharyngeal cancer.
